# Towards precise defect control in layered oxide structures by using oxide molecular beam epitaxy

**DOI:** 10.3762/bjnano.5.70

**Published:** 2014-05-08

**Authors:** Federico Baiutti, Georg Christiani, Gennady Logvenov

**Affiliations:** 1 Max-Planck Institute for Solid State Research, Heisenbergstrasse 1, D-70569, Stuttgart, Germany

**Keywords:** artificial superlattices, complex oxides, defect chemistry, interface effects, molecular beam epitaxy

## Abstract

In this paper we present the atomic-layer-by-layer oxide molecular beam epitaxy (ALL-oxide MBE) which has been recently installed in the Max-Planck Institute for Solid State Research and we report on its present status, providing some examples that demonstrate its successful application in the synthesis of different layered oxides, with particular reference to superconducting La_2_CuO_4_ and insulator-to-metal La_2−_*_x_*Sr*_x_*NiO_4_. We briefly review the ALL-oxide MBE technique and its unique capabilities in the deposition of atomically smooth single-crystal thin films of various complex oxides, artificial compounds and heterostructures, introducing our goal of pursuing a deep investigation of such systems with particular emphasis on structural defects, with the aim of tailoring their functional properties by precise defects control.

## Introduction

The progress in the synthesis of layered complex oxide compounds with high precision has been stimulated by the research on the many functional properties, from electrical to magnetic and optical, and on the multitude of structural and electronic phases that pertain to these strongly correlated materials [1]. Together with this, a major field of investigation is represented by interface effects occurring in oxides heterostructures [2]. In the last decades, their study has revealed the presence of unexpected properties, such as superconductivity [3–4], metallicity [5–6] and magnetism [7], which cannot be ascribed to any of the constituent phases taken singularly. Many, sometimes contradictory, mechanisms have been proposed in order to explain interface effects, including cationic intermixing [8], electronic reconstruction [9–10] and extrinsic doping [11–12], witnessing the complexity of the phenomena.

In order to achieve further progress in the study of complex oxides, there is an urgent need for a synergetic cross-fertilization of the chemistry and physics approaches. Recognizing this, researchers at the Max-Planck Institute for Solid State Research (MPI-FKF) have pursued a long-standing program to synthesize and investigate epitaxial metal oxide thin films and heterostructures based on the ALL-oxide MBE technique, which allows for the synthesis of structures of the best quality, together with the possibility to operate a compositional control at the most accurate level, possibly down to the single atomic layer [13]. The purpose is to provide new insights in the mechanisms underlying complex oxides and interfaces properties and to synthetize novel compounds and devices, giving particular importance to the role of defect chemistry in the definition of the functionalities and to the possibility of tuning them through a control of defects concentration and distribution.

### Oxide MBE

The MBE deposition technique is characterized by the evaporation of the elements constituting the desired compound from metal sources, followed by their recombination on the substrate surface. This provides the lowest kinetic energy of the incoming species, ensuring low undesired intermixing of different atoms or phases. Moreover, given the low deposition rate (few Ångstroms/minute), one can use appropriate monitoring tools to control the growth process very accurately. These two aspects represent major developments in comparison with the other most common oxide thin films deposition techniques, pulsed laser deposition (PLD) and sputtering. On the other hand, obvious limitations to the thickness of the samples and uncertainties in the deposition rates of each element, which eventually result in an off-stoichiometric growth, need to be taken into account. Indeed, at the present day there is no in-situ or ex-situ characterization method that can provide a control of the stoichiometry with an accuracy better than 1%. This makes the MBE method very uncertain, and only producing a large amount of samples ensures feasibility and reproducibility.

For the growth of complex oxides, one can use an MBE system whose construction is derived from the standard systems used for the synthesis of semiconducting heterostructures. The first modification required is the addition of a strong oxidation source. That is because one needs to work at low gas pressure (<10^−4^ Torr) in the growth chamber in order to maintain the “MBE regime” conditions characterized by a ballistic path of the atoms from the metal source to the deposition surface, while having at the same time the possibility to oxidize the evaporated metals to the desired valence state in a temperature range (usually between 600 °C and 700 °C), which is suitable for the growth of a highly crystalline structure. The feasibility of using ozone as a source for the successful growth of complex oxides and in particular high-*T*_c_ superconductors was demonstrated by several groups led by H. Mooij, A. Goldman, J. Eckstein, I. Bozovic, D. Schlom, M. Naito, T. Kawai, H. Koinuma, J.-P. Locquet and some others, and now the ozone delivery systems are commercially available and commonly implemented.

The specific conditions needed for the growth of complex oxides also require some further modifications. Since all parts operate under severe working conditions (high temperature, strongly oxidizing atmosphere), the materials for each component must be chosen extremely carefully. Materials such as molybdenum or PBN, which are often used in standard MBE, are not applicable in the oxide MBE given their tendency to form volatile oxides. Moreover, in order to synthesize a complex oxide, one needs an adequate number of evaporation sources. A modular design with vacuum gates between each source and the growth chamber is desired, enabling the opening of a source for the replacement of the material or for maintenance without venting the main growth chamber. Typically, two types of evaporation sources are used: thermal effusion cells (or Knudsen cell) and electron-beam sources. Thermal effusion cells can be heated up to 2000 °C and provide an extremely stable temperature and evaporating atomic flux. Electron-beam sources can be used for the evaporation of metals that require higher temperature (typically refractory metals), but in this case the atomic fluxes are less stable.

In the field of oxide MBE, a major development was represented by the layer-by-layer deposition scheme, called ALL-oxide MBE, introduced by the Varian group [14–16], which enables an extremely accurate control of the growth process and therefore a rational material design at various levels. In contrast to a standard MBE, in which all the constituents of the grown compound are evaporated together (codeposition), in this case the sources shutters are sequenced in a way that the correct number and species of atoms forming each atomic layer is placed on the growing surface at the right time, so that each of them is deposited singularly and in a sequence defined by the operator. Key tool for the ALL-MBE technique is the reflection high-energy electron diffraction (RHEED) system, which allows the in-situ characterization of the growth process, giving information about the morphology and the crystal structure of the film surface [17]. According to these, one can optimize the synthesis process, e.g., carry out corrections in the stoichiometry or in the growth conditions and control the sequence of the atomic layers.

In semiconductors MBE, the ALL-MBE method is used to deposit so called “delta doped” structures [18], where the dopants are confined to a single atomic plane. Extending this approach to the field of oxide MBE, one can do “single atomic layer engineering”, precisely defining the composition of each atomic layer, omitting or adding single layers to a given structure, stacking layers that belong to different compounds and designing artificial and metastable multilayers. A milestone in the ALL-oxide MBE technique was represented by the study of interface superconductivity between non-superconducting metallic La_1.55_Sr_0.45_CuO_4_ and insulating La_2_CuO_4_ layers, in which the replacement of a small amount of Cu by Zn in a single CuO_2_ plane allowed to localize the CuO_2_ plane responsible for this interface effect [19]. The ALL-oxide MBE technique has been recently used by A. Bhattacharya et al. to tailor the magnetic exchange interaction in LaMnO_3_–SrMnO_3_ [20], where magnetic properties of these superlattices were tuned between ferromagnetic and antiferromagnetic metallic states by inserting extra single-unit-cell layers of LaMnO_3_ and SrMnO_3_, respectively.

### The ALL-oxide MBE at the Max-Planck Institute for Solid State Research

**Figure 1:**
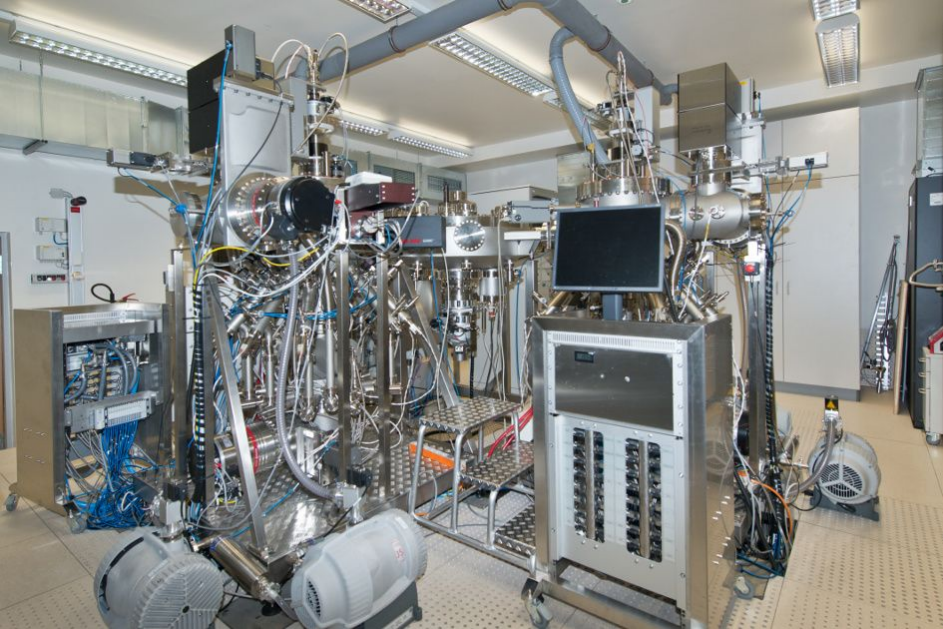
Photo of the dual chamber ALL-oxide MBE system installed in the Max-Planck Institute for Solid State Research.

The oxide MBE system that has been recently acquired by our institute is addressed to the atomic layer-by-layer growth of complex oxides and is equipped with two growth chambers, which are identical in construction and can work in parallel without interference, allowing us to increase the number of growth experiments and to reduce the time needed for the growth optimization. Designing and building were performed by DCA Instruments (Turku, Finland) according to the demands based on the experience achieved by of one of the authors during his work on the prototype ALL-oxide MBE systems at Oxxel GmbH (Bremen, Germany) and later in the Brookhaven National Laboratory (USA) [21]. The system has a cluster tool configuration. It is equipped with one ultra-high vacuum central distribution chamber (CDC) for the transfer of the samples. The CDC has eight connection ports: four of these are occupied by the two growth chambers, a load lock and a storage chamber, leaving four spare ports for a future expansion of the system. It will be possible to attach, for example, spectroscopic tools such as angular resolved photoemission spectroscopy (ARPES), time-of-flight ion scattering and recoil spectroscopy (TOF-ISARS), X-ray photoelectron spectroscopy (XPS) or any other, according to demands. Each chamber has a vertical design, which allows for the substrate transfer system to be fully automated. The substrate transfer between the load lock, the storage and each growth chamber is controlled by a PC-based logic controller. A photo of the MBE system is shown in Figure 1.

**Figure 2:**
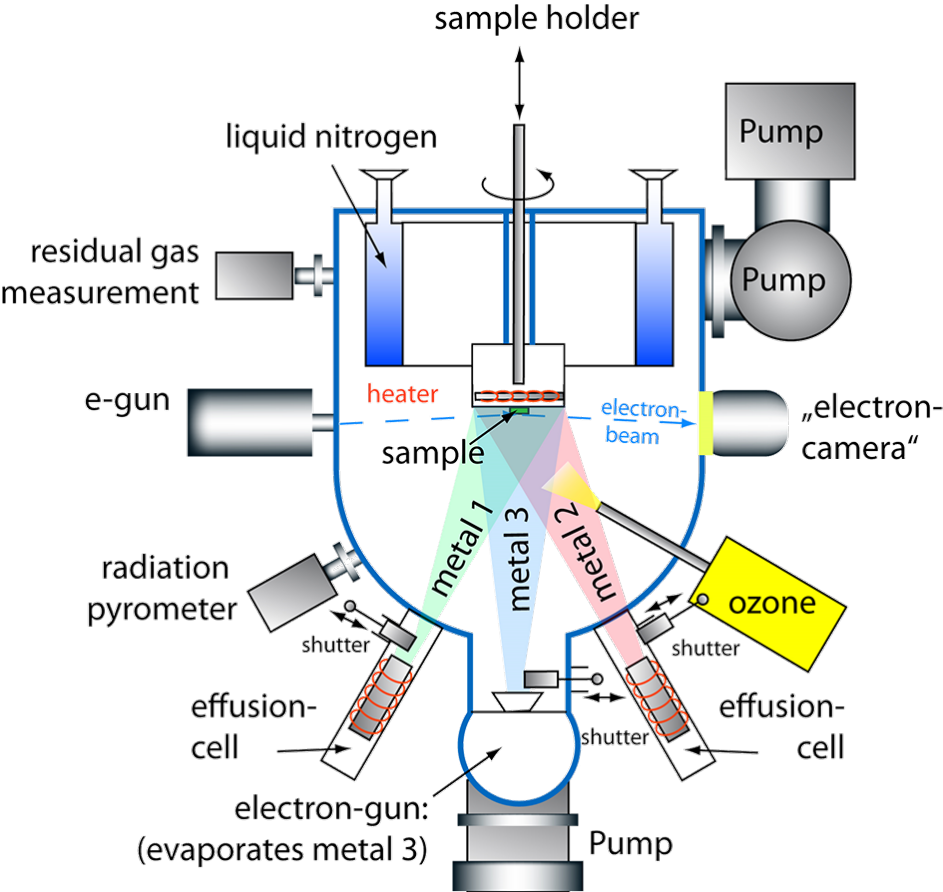
Sketch of the growth chamber.

The schematic view of a growth chamber is presented in Figure 2. It is equipped with ten water cooled spools, each of whom houses an elemental source, a computer-controlled linear-motion shutter, a gate valve and two ports for the future installation of an atomic absorption spectroscopy system to measure the deposition rates during the film growth process. The effusion cell ports are positioned symmetrically around the chamber center line. In addition, each growth chamber is equipped with a four pockets electron-beam evaporator directly located below the substrate position. The growth chamber is provided with an internal liquid nitrogen cooled cryogenic panel surrounding the substrate area, and its geometry is optimized for a maximum substrate size of 3″. The substrate manipulator has motorized rotation and vertical translation. Pumping is provided by a corrosive gas version of a turbo-pump with a scroll-type backing pump and by an ion-pump. A differential pumping module is used as a common chamber, connected to all effusion cells and the electron gun differential pumping lines. This way, each source can be opened for service and replacement of a source element without venting the growth chamber within a short period of time, even during the film growth. The differential pumping module is also used to pump the electron gun of the RHEED system and the load locked quartz crystal monitor (QCM) head. The QCM, which is mounted on a linear bellows assembly and is separated from the growth chamber by a gate valve, is used to calibrate the absolute deposition rates for each source before starting the process, while the RHEED system allows for the in-situ monitoring of the growth in real time. The oxygen resistive heater provides a maximum temperature of the heater element up to 1200 °C and the substrate temperature is controlled by a radiation pyrometer. The oxidation during the film growth is ensured by the delivery of pure ozone. Distilled ozone is collected from an ozone generator system and stored in an insulated still, where it is absorbed by silica gel spheres. The still temperature is adjusted by means of liquid nitrogen cooling and a heating element in order to control the evaporation rate of ozone, which is delivered to the growth chamber. The typical pressure in the growth chamber during a deposition is 10^−6^ to 10^−5^ Torr. The accurate layer-by-layer deposition control is enabled by electro-pneumatic linear shutters positioned in front of each source, which accurately control the amount of atoms deposited for each species and each layer. The shuttering system is implemented in a controlling software system that allows the operator to write recipes defining the composition of each atomic layer, to modify them during the film growth and to adjust the stoichiometry according to the RHEED pattern.

## Results and Discussion

**Figure 3:**
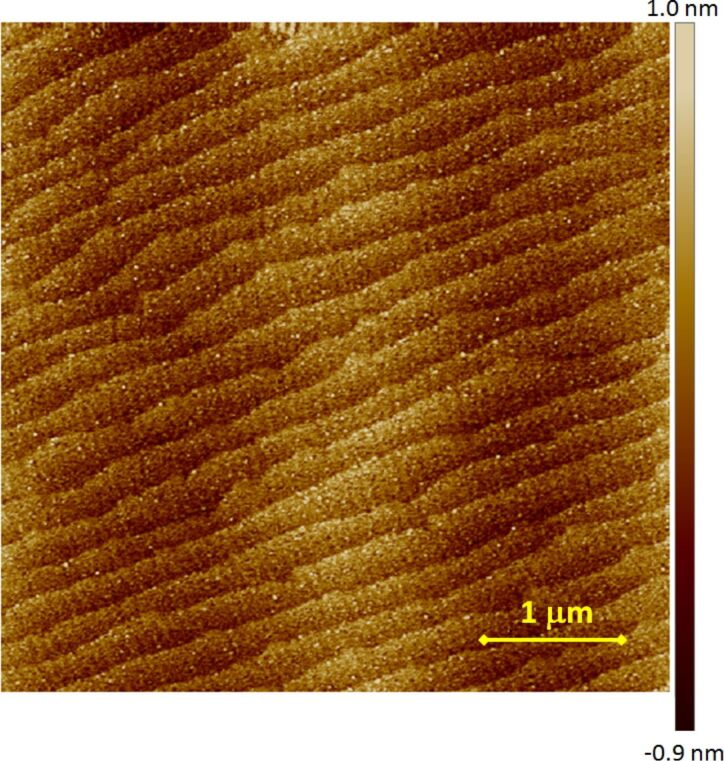
AFM image of 25 nm thick La_2_NiO_4_ film on STO substrate.

**Figure 4:**
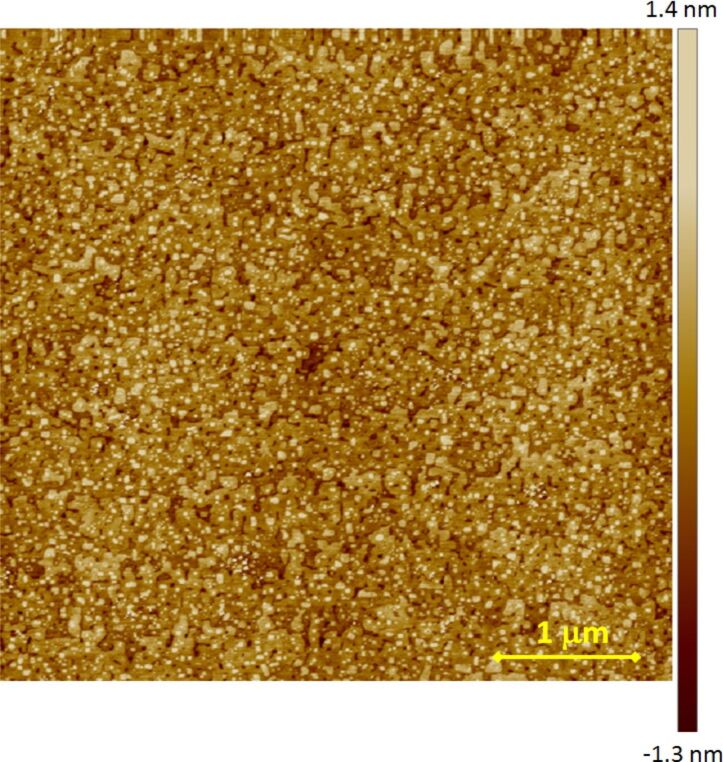
AFM image of 25 nm thick La_2_CuO_4_ film on LSAO substrate.

Using our ALL-oxide MBE system we can deposit a broad range of thin single phase oxide films and heterostructures. During the system optimization phase, we have experimented with La_2−_*_x_*Sr*_x_*CuO_4_ and La_2−_*_x_*Sr*_x_*NiO_4_ with different doping levels (*x*), LaNiO_3_, LaAlO_3_, LaSrAlO_4_ and several other complex oxides. In particular, we have deposited superlattices with ultrathin layers, down to one unit cell thick. In most cases, such films and heterostructures have good cristallinity, smooth surfaces and interfaces. The crystallographic structure of our films is determined by high-resolution X-Ray diffraction (XRD) using a 4 circles X-ray machine by Bruker, while surface morphology is measured by a multimode atomic force microscope (AFM) by Veeco. In Figure 3 and Figure 4 we show AFM images for 25 nm thick La_2_NiO_4_ on SrTiO_3_ (STO) substrate and for La_2_CuO_4_ on LaSrAlO_4_ (LSAO) grown in our system, that suggest how our films are free from secondary phases outgrows and that layer-by-layer growth mode is achieved. The STO substrate was TiO_2_ terminated by etching in buffered HF acid and a following annealing at 950 °C in oxygen flow. The LSAO substrate was simply cleaned in an ultrasonic bath with aceton and following isopropanol without special treatment for surface termination. Thus, we suppose that the LSAO substrate has a mixed termination of LaSrO and AlO_2_ layers. The root mean square roughness is in both cases *R*_ms_ = 0.4 nm over a scanning area of 25 μm^2^. In the former image, one can see clear atomic terraces, about 300 nm wide, with 0.5 unit cell tall steps due to the slight miscut angle of the STO substrate (<0.1°). The AFM image of the La_2_CuO_4_ film on LSAO does not clearly exhibit such atomic terraces first because, according to specifications, the miscut angle is less than 0.01°, which would lead to an atomic terraces width larger than 1.2 µm, secondarily because the mixed termination of the substrate can act in the direction of smearing these terraces given the fact that the surface roughness of each of the terraces is comparable to half of the La_2_CuO_4_ molecular layer size of about 0.65 nm.

**Figure 5:**
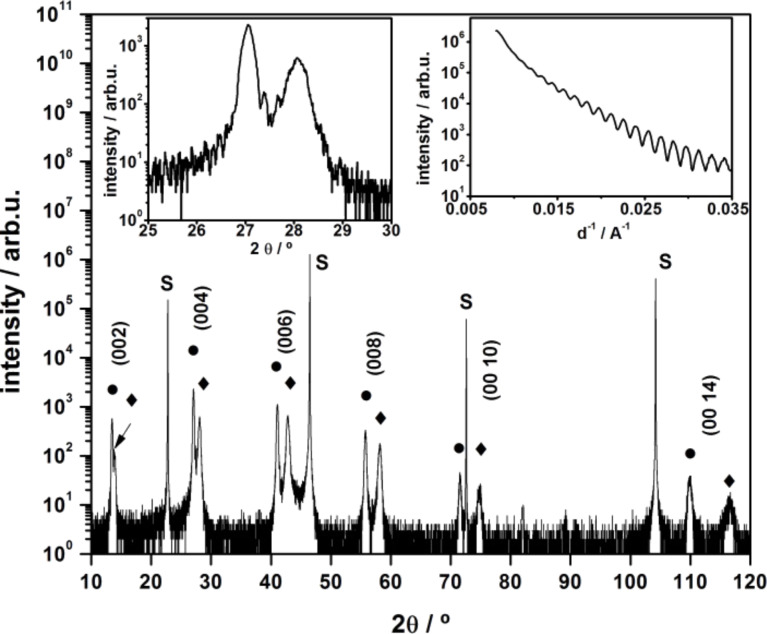
XRD θ–2θ scan of a bilayer heterostructure La_2_NiO_4_ (diamonds)–La_2_CuO_4_ (circles) on a STO(100) substrate (S). The thickness of the La_2_NiO_4_ and La_2_CuO_4_ is 26 nm and 40 nm respectively, according to the number of RHEED oscillations recorded during the growth. In the left inset: magnification of the (004) diffraction peak; in the right inset: XRR scan.

The XRD θ–2θ scan for a La_2_NiO_4_–La_2_CuO_4_ bi-layer is shown in Figure 5. The high quality crystal structure of the epitaxial layers is confirmed by the observation of pronounced separate peaks from La_2_NiO_4_ and La_2_CuO_4_ layers up to (00 14) Bragg reflections, by the presence of Laue fringes around the main diffraction peaks (left inset) and by the X-ray reflectance (XRR) oscillations observed at low grazing angle (right inset). No XRR modulations deriving from the bilayer structure are expected, given the similar density of the constituent layers (7.22 g/cm^3^ for LCO and 7.14 g/cm^3^ for LNO).

**Figure 6:**
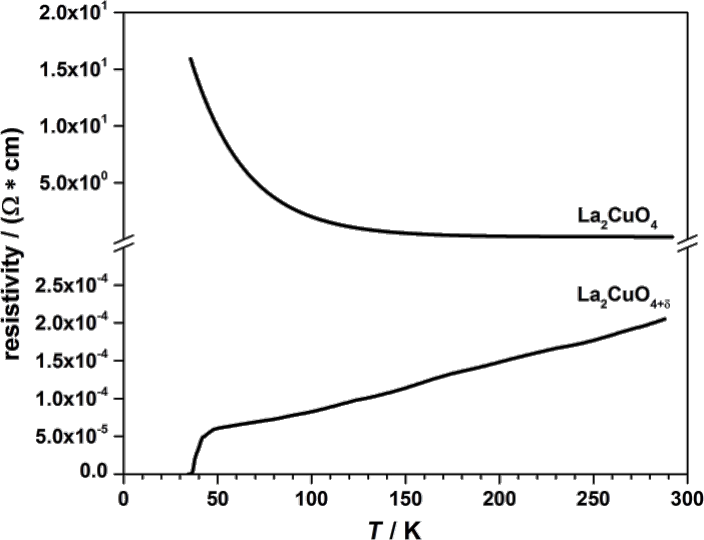
Resistivity versus temperature for the parent compound La_2_CuO_4_ and for the superconducting La_2_CuO_4+δ_.

A crucial parameter for the growth of complex oxides is the system oxidation power. The oxidation state of the transition metals in the ozone-assisted MBE strongly depends on the ozone concentration in the growth chamber, that is, the efficiency of the ozone delivery, as the ozone molecules lifetime is in the range of some seconds. To our knowledge, one of the most demanding tests to check if the optimal concentration of ozone is reached is the synthesis of superconducting cuprates thin films [22]. To realize this test we tried to synthesize superconducting La_2_CuO_4_ epitaxial films. This parent compound for the La_2−_*_x_*Sr*_x_*CuO_4_ family is an antiferromagnetic insulator that can be converted to superconducting La_2_CuO_4+δ_, with *T*_c_ ≈ 40 K, upon annealing in ozone atmosphere due to the introduction of interstitial oxygen [23]. In Figure 6, we present resistivity versus temperature curves for two La_2_CuO_4_ films grown in our lab, carried out by using a four-probe method with Pt contacts deposited by sputtering on the corners implemented in a custom-built DC measurements setup. Both films were grown under the same conditions: substrate temperature 620 °C and chamber pressure 2.5·10^−5^ Torr. The difference was in the cooling process after the growth. The insulating film was cooled down to 200 °C with a cooling rate of 30 °C/min and then the ozone delivery was stopped and the film was cooled down to room temperature in vacuum (10^−9^ Torr), and shows a resistivity curve that is an exponential function of temperature, while the second one, cooled down to room temperature at an increased ozone pressure of 5·10^−5^ Torr, shows a superconducting transition around 40 K. This way we found that, in our growth conditions, (i) copper is oxidized to the maximum valence state Cu^2+^, which is the first prerequisite to form the La_2_CuO_4_ phase and (ii) the ozone partial pressure in our deposition system is adequate to provide the optimal doping level.

**Figure 7:**
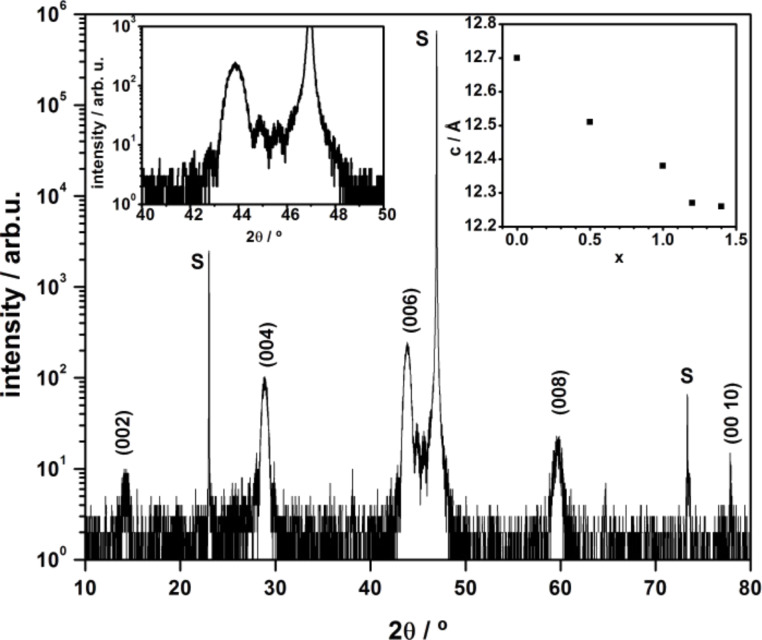
XRD θ–2θ scan of 13 nm thick La_1_Sr_1_NiO_4_ on LSAT(100) (S). Laue fringes can be seen in the left inset, magnification around the (006) diffraction peak. In the right inset, the c-axis value, obtained by applying the Bragg equation for each peak position and subsequent refinement with the Nelson–Riley function, for thin films of La_2−_*_x_*Sr*_x_*NiO_4_ epitaxially grown on LSAT(100) substrate.

**Figure 8:**
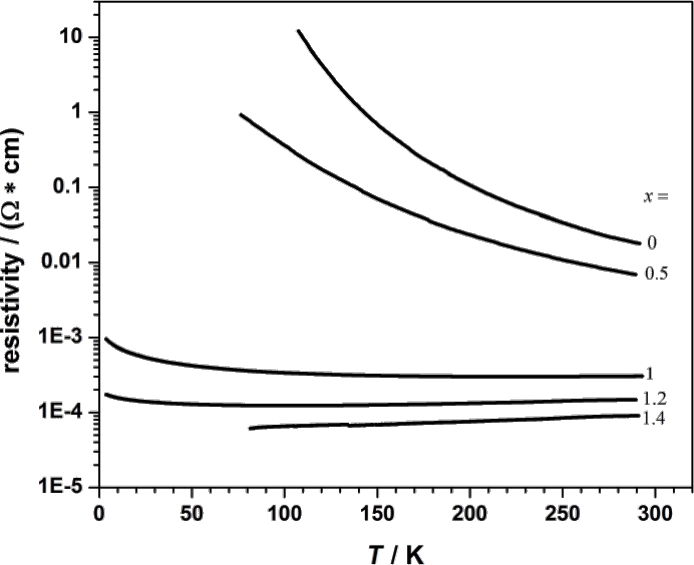
Resistivity versus temperature for La_2−_*_x_*Sr*_x_*NiO_4_ with different doping levels.

Using our ALL-oxide MBE system, we also focused on the growth of lanthanum nickelate La_2−_*_x_*Sr*_x_*NiO_4_ with different Sr doping levels (*x*). This class of oxides is interesting because it is, under many aspects of crystal chemistry, similar to the superconducting La_2−_*_x_*Sr*_x_*CuO_4_. The transition metal Nickel (Ni) can have several valence states, Ni^1+^, Ni^2+^ and Ni^3+^. Since La_2_NiO_4_ is one of the Ruddlesden–Popper phases A*_n_*_+1_B*_n_*O_3_*_n_*_+1_ with *n* = 1, it is challenging to grow it as a single phase without the intergrowth of other phases. Here we report that, by using our ALL-oxide MBE, we synthesized atomically smooth La_2−_*_x_*Sr*_x_*NiO_4_ epitaxial films with 0 ≤ *x* ≤ 1.4. Diffraction measurements show that our films are *c*-axis oriented single phases, as can be seen in Figure 7, where the XRD θ–2θ scan for a composition with the nominal doping level *x* = 1 is shown. In the right inset, we report the calculated *c*-axis values for the different grown compositions: they exhibit a monotonical decrease for increasing doping content. The in-plane resistivity versus temperature for La_2−_*_x_*Sr*_x_*NiO_4_ with different Sr doping level is shown in Figure 8. The resistivity value systematically decreases with increasing Sr content. All the experimental data are in good agreement with those reported previously for La_2−_*_x_*Sr*_x_*NiO_4_ epitaxial thin films grown by PLD [24]. Further analyses of films crystal structure, including high-resolution transmission electron microscopy (HR-TEM) and transport properties and will be presented elsewhere [25].

## Conclusion

We described our ALL-oxide MBE system, equipped with the state-of-art technology, a high level of automation and modularity and we showed some of its capabilities in the growth of different complex oxides and heterostructures. No alternative deposition techniques can provide a comparable level of flexibility and control at the sub-unit cell level, and we aim to take advantage of it when synthesizing various combinations and sequences of different layered oxides, trying to induce novel ground states in the interfaces or in the oxides blocks and to define innovative model systems for physical and chemical investigations. As previously mentioned, though, limitations occur, including for example the range of metal sources that can be evaporated, the oxidation power of the system and the control of stoichiometry during the growth process.

Besides these, other aspects have to be considered. First to be cited is the presence of the substrate whose roughness is in the best of the cases maintained, not smoothed, during the growth process, and which acts affecting layers morphology and interface abruptness. In addition, heteroepitaxy between substrate and film induces a strain state, which is responsible for the modification of the functional properties of the compound. By using the ALL-oxide MBE capabilities, in combination with advanced ex-situ investigation tools such as HR-TEM, we could deeper investigate how the epitaxial strain state, both in single phase films and in superlattices, is localized and how it is related to the functional and the structural properties. Another crucial point is given by thermodynamical limitations. Indeed, one should consider that the synthesis of artificial compounds or interfaces can lead to structural instabilities due, for instance, to solubility limits or to the tendency of atoms intermixing at interfaces resulting in cationic redistribution or intergrowth of secondary phases. Moreover, how do charge compensation mechanisms act at interfaces? What is the result of the “polar discontinuity” and of the presence of interfacial space charge zones? Together with electronic and cationic rearrangement, one should consider the major role which can be played by oxygen defects, which can easily be formed and migrate in the structure.

Taking all this into account, our approach will be based on the careful investigation of the aspects related to defects formation and distribution, with the aim to put in evidence their crucial role in the definition of the functionalities of complex oxide heterostructures. Taking advantage of this investigation, we aim to move from the idea of a “perfect sample”, which resembles theoretical models with minimized presence of imperfections, to the concept of precise defects control, based on the fine tuning of defects concentration and distribution. Its application to the “layer by layer engineering” by ALL-oxide MBE, could allow us to develop new methods for the fabrication of layered oxides and heterostructures with novel functional properties.

## References

[1] Tsymbal E Y, Dagotto E R A, Eom C-B (2012). Multifunctional oxide heterostructures.

[2] Hwang H Y, Iwasa Y, Kawasaki M, Keimer B, Nagaosa N, Tokura Y (2012). Nat Mater.

[3] Reyren N, Thiel S, Caviglia A D, Kourkoutis L F, Hammerl G, Ricter C, Schneider C W, Kopp T, Rüetschi A S, Jaccard D (2007). Science.

[4] Gozar A, Logvenov G, Kourkoutis L F, Bollinger A T, Giannuzzi L A, Muller D A, Bozovic I (2008). Nature.

[5] Ohtomo A, Hwang H Y (2004). Nature.

[6] Bhattacharya A, May S J, te Velthuis S G E, Warusawithana M, Zhai X, Jiang B, Zuo J M, Fitzsimmons M R, Bader S D, Eckstein J N (2008). Phys Rev Lett.

[7] Takahashi K S, Kawasaki M, Tokura Y (2001). Appl Phys Lett.

[8] Willmott P R, Pauli S A, Herger R, Schlepütz C M, Martoccia D, Patterson B D, Delley B, Clarke R, Kumah D, Cionca C (2007). Phys Rev Lett.

[9] Wu J, Pelleg O, Logvenov G, Bollinger A T, Sun Y-J, Boebinger G S, Vanević M, Radović Z, Božović I (2013). Nat Mater.

[10] Nakagawa N, Hwang H Y, Muller D A (2006). Nat Mater.

[11] Kalabukhov A, Gunnarsson R, Börjesson J, Olsson E, Claeson T, Winkler D (2007). Phys Rev B.

[12] Di Castro D, Salvato M, Tebano A, Innocenti D, Aruta C, Prellier W, Lebedev O I, Ottaviani I, Brookes N B, Minola M (2012). Phys Rev B.

[13] Schlom D G, Haeni J H, Lettieri J, Theis C D, Tian W, Jiang J C, Pan X Q (2001). Mater Sci Eng, B.

[14] Eckstein J N, Bozovic I (1995). Annu Rev Mater Sci.

[15] Bozovic I, Eckstein J N, Klausmeier-Brown M E, Virshup G F (1992). J Supercond.

[16] Chaiken A, Wall M A, Howell R H, Bozovic I, Eckstein J N, Virshup G F (1996). J Mater Res.

[17] Bozovic I, Eckstein J N (1995). MRS Bull.

[18] Schubert E F (1990). J Vac Sci Technol, A.

[19] Logvenov G, Gozar A, Bozovic I (2009). Science.

[20] Santos T S, Kirby B J, Kumar S, May S J, Borchers J A, Maranville B B, Zarestky J, te Velthuis S G E, van den Brink J, Bhattacharya A (2011). Phys Rev Lett.

[21] Logvenov G, Bozovic I (2008). Physica C.

[22] Schlom D G, Marshall A F, Sizemore J T, Chen Z J, Eckstein J N, Bozovic I, Von Dessonneck K E, Harris J S, Bravman J C (1990). J Cryst Growth.

[23] Bozovic I, Logvenov G, Belca I, Narimbetov B, Sveklo I (2002). Phys Rev Lett.

[24] Shinomori S, Okimoto Y, Kawasaki M, Tokura Y (2002). J Phys Soc Jpn.

[R25] 25Baiutti, F. et al. To be published.

